# The IPASC data format: A consensus data format for photoacoustic imaging

**DOI:** 10.1016/j.pacs.2022.100339

**Published:** 2022-02-26

**Authors:** Janek Gröhl, Lina Hacker, Ben T. Cox, Kris K. Dreher, Stefan Morscher, Avotra Rakotondrainibe, François Varray, Lawrence C.M. Yip, William C. Vogt, Sarah E. Bohndiek, Members of the International Photoacoustic Standardisation Consortium (IPASC)

**Affiliations:** a Cancer Research UK Cambridge Institute, University of Cambridge, Cambridge, United Kingdom; b Department of Physics, University of Cambridge, Cambridge, United Kingdom; c Department of Medical Physics and Biomedical Engineering, University College London, London, United Kingdom; d German Cancer Research Center, Division of Computer Assisted Medical Interventions, Heidelberg, Germany; e Heidelberg University, Faculty of Physics and Astronomy, Heidelberg, Germany; f iThera Medical GmbH, Munich, Germany; g Univ Lyon, INSA-Lyon, Université Claude Bernard Lyon 1, CNRS, Inserm, CREATIS UMR 5220, U1294, F-69621, Lyon, France; h Department of Medical Biophysics, Schulich School of Medicine and Dentistry, Western University, London, Canada; i Imaging Program, Lawson Health Research Institute, London, Canada; j Center for Devices and Radiological Health, US Food and Drug Administration, Silver Spring, MD, United States

**Keywords:** Data format, Photoacoustic imaging, Open science, Standardisation, Metadata

## Abstract

Photoacoustic imaging (PAI) is an emerging modality that has shown promise for improving patient management in a range of applications. Unfortunately, the current lack of uniformity in PAI data formats compromises inter-user data exchange and comparison, which impedes: technological progress; effective research collaboration; and efforts to deliver multi-centre clinical trials. To overcome this challenge, the *International Photoacoustic Standardisation Consortium* (IPASC) has established a data format with a defined consensus metadata structure and developed an open-source software application programming interface (API) to enable conversion from proprietary file formats into the IPASC format. The format is based on Hierarchical Data Format 5 (HDF5) and designed to store photoacoustic raw time series data. Internal quality control mechanisms are included to ensure completeness and consistency of the converted data. By unifying the variety of proprietary data and metadata definitions into a consensus format, IPASC hopes to facilitate the exchange and comparison of PAI data.

## 1 Introduction

Translation of photoacoustic imaging (PAI) into clinical trials has found a diverse landscape of potential applications for the technology, which unites the high contrast afforded by optical excitation with the resolution and penetration depth available through ultrasound detection [1], [2], [3], [4]. PAI can be naturally applied across a range of length scales due to the broadband nature of photoacoustic waves, with systems ranging from microscopic resolution (at sub-millimetre imaging depth) to macroscopic, often tomographic, configurations (up to centimetres imaging depth). Such diversity in system configurations enables application-specific customisation yet introduces complexity in data acquisition and management. In all cases, however, the physics of the photoacoustic effect and thus the overall nature of the data are the same. For these reasons, the algorithms used for data processing, image reconstruction and data display, as well as the respective metadata, have much in common. Despite these similarities, there is at present no community consensus on a data format for storing and sharing PAI data.

The value of a standardised data format has been recognised in other imaging communities, leading to the developments of tailored formats such as the brain imaging data structure (BIDS) for magnetic resonance imaging (MRI) [5], Neuroimaging Informatics Technology Initiative (NIfTI) format for neuroimaging [6], the Shared Near-Infrared Spectroscopy Format (SNIRF) for functional near-infrared-spectroscopy [7] or the ultrasound file format for ultrasound (UFF) [8]. The Digital Imaging and Communications in Medicine (DICOM) format is the current international standard for handling clinical imaging datasets [9], but the format is designed to store reconstructed images only and targeted towards clinical use.

The lack of a standardised format for raw PAI time series data impedes access, exchange and usability of acquired data between different researchers, vendors and organisations. A standard photoacoustic data format would have two substantial benefits. First, it would assist those working with photoacoustic data on a day-to-day basis by facilitating better data handling. For example, it could aid in comparison of data obtained from different sites or using instruments from different vendors. Second, a standard data format, if widely adopted, would help those developing photoacoustic technologies and visualisation software to present a united front to clinicians, data scientists, and other potential users. For example, a common format could accelerate the development and testing of new image reconstruction and processing methods by making PAI data more transparent.

Nonetheless, the development of a standardised format for PAI is associated with challenges. As highlighted above, system configurations for PAI are diverse, leading to a wide variability in the available (and relevant) metadata between systems. Moreover, time series data can be large in size and high-dimensional. Additionally, image acquisition parameters that can be highly relevant for post-processing algorithms may be unavailable (for example, because of hardware constraints or intellectual property protection) or subject to uncertainties.

The development, introduction, and adoption of a standard file format relies heavily on the widespread participation of researchers within the community. The International Photoacoustic Standardisation Consortium (IPASC) is a community-led effort committed to assisting and supporting the introduction of standards within the field of PAI. The work of IPASC is streamlined into three working groups, covering: study design; phantom development; and data acquisition and management (DAM). The DAM working group has taken up the task of developing a standardised way to store PAI data in a tailored digital format. The format underwent several optimisation procedures [10] in which feedback from the PAI community was collected and used to improve content and structure of the format. Here, these combined efforts are presented. First, relevant terminology and definitions are introduced. Second, the structure of the IPASC format and its associated metadata are highlighted. Finally, the organisation and workflow of an open-source application programming interface (API) for data conversion are presented. By introducing this format, IPASC hopes to facilitate PAI data handling and processing, thereby supporting future standards development within the PAI community and accelerating efforts in clinical translation.

## 2 Terminology and definitions

This section provides definitions of terms to avoid ambiguity. For further details, please refer to the IPASC ‘Terms and Definitions’ [11] and ‘Photoacoustic Data and Device Parameters’ consensus documents [12] that have been adopted by IPASC and are available on the IPASC website.

**Raw Time Series Data**: A *time series* refers to the time-sampled signal from one detection element. *Raw time series data* refers to an unaltered set of such time series, one for each detection element.

**Device**: A specific make and model of a photoacoustic device (hardware and/or software). This may include lab prototypes, systems sold for research use, or medical devices regulated in the United States by the Food and Drug Administration (FDA) and in the European Union (EU) by CE marking [13]. A distinction is drawn between **full-scan** (image acquisition without sequential movement of illumination/detection array or target) and **composite-scan** devices (image acquisition by sequential change of position and/or orientation of illumination/detection array or target).

**Modality**: A category of imaging device, characterised by a distinct physical principle [13] (e.g. PAI).

**Measurement**: A set of raw time series data corresponding to a single illumination and acquisition step of the imaging device. For full-scan devices, only one measurement is taken, whilst for composite-scan devices, multiple measurements contribute to the same image. For composite scans, the *Measurement Spatial Pose* datum can be used to assign the relative spatial pose of a measurement.

**Image**: An array of values varying in two or more spatial dimensions derived from analysis of an imaging signal and corresponding to an array of spatial locations in the imaged object [13]. In the context of this document, a photoacoustic image refers to the result of mapping raw time series data into the spatial domain. An image can be derived from one or multiple *measurements*. An image might be subject to post-processing steps, such as envelope detection, display dynamic range, thresholding, bit depth discretisation, gain adjustment, colour mapping, or fluence correction.

**Detection Element**: A specific material element capable of converting mechanical energy to electrical energy and in some cases converting electrical energy to mechanical energy [14]. Here, it refers to e.g. a piezoelectric crystal or a laser interferometer.

**Illumination Element**: A specific material element capable of emitting light to illuminate a target, e.g. an optical fibre that conveys the light generated by a laser or light-emitting diode (LED) source. All defined illumination elements must be fixed throughout the entirety of the acquisition of one measurement.

## 3 The IPASC data format

A PAI dataset consists typically of one (or more) images representing the projection of a target volume onto an image plane (two-dimensional (2D) imaging), a series of images representing volume (three-dimensional (3D) imaging), or multiple acquisitions of the same 2D or 3D dataset over time and/or wavelength to produce a dynamic series of acquisitions (multi-dimensional imaging). The IPASC data format is designed to store measurements as raw time series data and not as reconstructed images to prevent loss of information and decrease the complexity of the data format. The raw time series data are accompanied by relevant metadata to enable reproducible reconstruction.

To accommodate the varied nature of PAI datasets, the Hierarchical Data Format (HDF5) [15] was chosen as the data format due to the following benefits: HDF5 (1) is able to store and organise large amounts of data within a single file container; (2) is platform-independent; (3) supports an unlimited variety of data types; (4) is widely used among the scientific community; (5) is openly available; (6) implements a high-level API with, for example, MATLAB,^4^ Python [16], C++,^5^ or Java interfaces;^6^ and (6) contains the descriptive metadata within the data file, so metadata cannot get lost when exchanging files. HDF5 uses two objects types: datasets, which are multidimensional arrays of a homogeneous type, and groups, which are container structures holding datasets and other groups. In the IPASC data format, metadata are stored in the form of user-defined, named attributes attached to groups and datasets.

## 4 Metadata attributes

Metadata provide information about relevant aspects of data. Within the IPASC data format, each metadatum is characterised by a series of attributes to describe and define its use and boundary conditions (Table 1). If applicable, further specifications by nested attributes are given. All units of the metadata are given in the International System of Units (SI units) unless otherwise specified.

The metadata of the IPASC data format are organised into three sub-categories: (1) Minimal Metadata, (2) Acquisition Metadata and (3) Device Metadata. Whilst the format tries to accommodate the most relevant metadata parameters for full PAI data description, the wide variety of existing systems and constant technological progress may require the addition of further parameters that have not been considered yet in our current framework. To account for this need, the format allows for the addition of custom parameters by the end user, thereby providing the flexible structure that is required to accommodate the diverse nature of PAI data.

**Table 1:** Overview of the metadata attributes used to describe each of the metadata items within the IPASC format.

Metadata attributes	
Necessity	Can be *‘Minimal’* or *‘Report if present’*.
dtype	Data type of the attribute.
Units	SI unit of the attribute if applicable.
Description	A short description of the attribute.
Method name	The name of the method or function that can be called in a programming language in order to obtain the information of this attribute.
Condition	Constraints of the attribute, if applicable.
Nested attribute	A sub-attribute that further describes an attribute.
Measurement device	A specific type of a nested attribute that further describes measurement device details if required.
Measurement device type:	A string literal describing the measurement device for this attribute, e.g. ‘pyroelectric sensor’ or ‘wavemeter’.
Measurement device manufacturer:	A string literal describing the manufacturer of the measurement device, e.g. ‘Thorlabs’.
Measurement device serial number:	A string literal comprising the serial number of the measurement device.
Calibration date:	A timestamp referring to the date when the measurement device was last calibrated.

**(1) Minimal Metadata**

*Minimal Parameters* are indicated by the *Necessity* attribute and comprise all parameters that are required to read PAI data and reconstruct any image from the raw time series data. Any additional information should be reported in the metadata if available. The minimal parameter set contains:

**Container Format Metadata:** The inherent features of the file format, which specify mandatory parameters. They include the unique universal identifier (UUID), the type of compression, and the type of encoding.

**Binary Data Metadata:** The metadata that make the binary data machine-readable. They include specifications on data type, dimensionality, and the size of each dimension.

**A/D (Analogue/Digital) Sampling Rate:** A single value referring to the rate at which samples of the analogue signal are taken to be converted into digital form.

**Acquisition Wavelengths:** A 1D array that contains all wavelengths used for the image acquisition.

**Detector Positions:** The positions of each detection element in 3D Cartesian coordinates [x1, x2, x3].

**Field of View:** An array defining an approximate cuboid (3D) area that should be reconstructed in 3D Cartesian coordinates [x1${}_{\text{start}}$, x1${}_{\text{end}}$, x2${}_{\text{start}}$, x2${}_{\text{end}}$, x3${}_{\text{start}}$, x3${}_{\text{end}}$]. A 2D *Field of View* can be defined by setting the start and end coordinate of the respective dimension to the same value.

**(2) Acquisition Metadata**

**Fig. 1:**
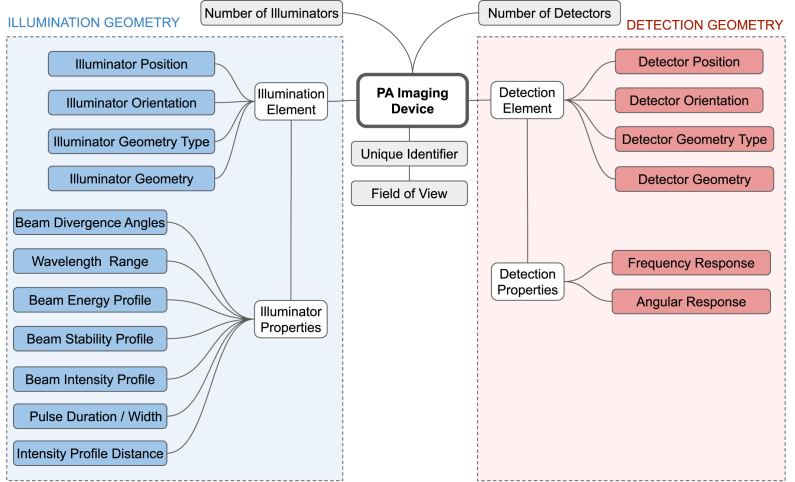
Overview of the *Device Metadata* parameters. In this representation, a device is modelled to have a number of detection elements and illumination elements that each have characteristic properties.

The *Acquisition Metadata* describe the acquisition settings at data capture. In addition to the *Minimal Metadata*, they include the following parameters:

**Regions of Interest:** A list of named regions within the underlying 3D Cartesian coordinate system (cf. *Device Metadata*). Strings containing the region names are mapped to arrays that define either an approximate cuboid area (cf. *Field of View*) or a list of coordinates describing a set of 3D Cartesian coordinates surrounding the named region.

**Photoacoustic Imaging Device Reference:** A string referencing the UUID of the PAI device description as defined in the *Device Metadata*.

**Pulse Energy:** A value specifying the pulse energy used to generate the photoacoustic signal. If the pulse energies are averaged over many pulses, the average value must be specified.

**Measurement Timestamps:** An array specifying the time at which a measurement was recorded.

**Measurement Spatial Pose:** Coordinates describing the position and orientation changes of the acquisition system relative to the measurement of reference (first measurement).

**Time Gain Compensation:** An array containing relative factors that have been used to correct the time series data for the effect of acoustic attenuation.

**Overall Gain:** A single value describing a factor used to modify the amplitude of the raw time series data.

**Element-dependent Gain:** An array that contains the relative factors used for apodisation or detection element-wise sensitivity corrections.

**Temperature Control:** An array describing the temperature of the imaged space (covering both the imaged medium and the coupling agent) for each measurement.

**Acoustic Coupling Agent:** A string representing the acoustic coupling agent that is used.

**Speed of Sound:** Either a single value representing the mean global speed of sound in the entire imaged medium or a 3D array representing a heterogeneous speed of sound map in the device coordinate system. This definition covers both the imaged medium and the coupling agent.

**Scanning Method:** A string representing the scanning method that is used. The following descriptions can be used: (“composite_scan”, “full_scan”). This flag determines the way the metadatum “measurement” is defined.

**Measurements Per Image:** A single value describing the number of measurements that constitute the dataset corresponding to one image.

**Frequency Domain Filter:** An array defining the frequency threshold levels that are applied to filter the raw time series data, containing [lower, higher] −3 dB points of the filter in Hertz. [lower, −1] denotes a high-pass filter and [−1, higher] denotes a low-pass filter.

**(3) Device Metadata**

The *Device Metadata* carry all information necessary to describe a PAI device (Fig. 1). In contrast to the *Acquisition Metadata*, they aim to facilitate modelling of how individual systems acquire data, for example, to enable corrections for directivity, or the application of illumination geometry-specific fluence correction algorithms, such as described in [17] or [18]. By collecting these metadata, a database of commercial and custom-built PAI devices can be created with all parameters necessary to construct a digital twin of the imaging device hardware. Each system is assigned with a unique identifier, which is referred to in the recorded photoacoustic data. The *Device Metadata* include:

**Universally Unique Identifier:** A randomly generated hexadecimal string that can be used to reference the device.

**Field of View:** Coordinates describing an approximate cuboid of the area detectable by the PAI device.

**Number of Illumination Elements:** The number of illuminators used in the PAI device.

**Number of Detection Elements:** The number of transducer elements used in the PAI device.

***Illumination Element***

**Illuminator Position:** Coordinates defining the position of the illuminator centroid.

**Illuminator Orientation:** Coordinates defining the direction unit vector of the illuminating beam.

**Illuminator Geometry Type:** A string describing the shape of the optical fibre (bundle) output.

**Illuminator Geometry:** Values defining the numerical geometry of the optical fibre (bundle) output. The data type and content of this metadatum are determined by the *illuminator geometry type* field.

**Wavelength Range:** An array of three values [minimum wavelength, maximum wavelength, accuracy] describing the wavelengths that can be generated by the illuminator.

**Laser Energy Profile:** An array of two double arrays [wavelengths, energies] which describes the laser energy of the illuminator.

**Laser Stability Profile:** An array of two double arrays [wavelengths, standard deviations] representing the standard deviation of the pulse-to-pulse laser energy of the illuminator.

**Pulse Duration/Width:** A value describing the total length of a laser pulse (measured as the time interval between the half-power points on the leading and trailing edges of the pulse.)

**Beam Intensity Profile:** Array of two double arrays [positions, intensities] specifying the relative laser beam intensity according to the planar emitting surface of the illuminator at the distance defined in *intensity profile distance*.

**Intensity Profile Distance:** An array describing the distance from the light source for measuring its *beam intensity profile*.

**Beam Divergence Angles:** A value specifying the opening angle of the laser beam with respect to the orientation vector.

***Detection Element***

**Detector Position:** Coordinates defining the position of the detection element centroid.

**Detector Orientation:** Coordinates defining the direction unit vector of the detector.

**Detector Geometry Type:** A string describing the type of detector geometry.

**Detector Geometry:** Values defining the numerical geometry of the detector. The data type and content of this metadatum are determined by the *detector geometry type* field.

**Frequency Response:** An array of two values [center frequency, bandwidth] characterising the frequency response of the detection element towards the incident pressure waves.

**Angular Response:** An array of two values [incident angle, response] characterising the angular sensitivity of the detection element to the incident angle (relative to the element’s orientation) of the incoming pressure wave.

**Fig. 2:**
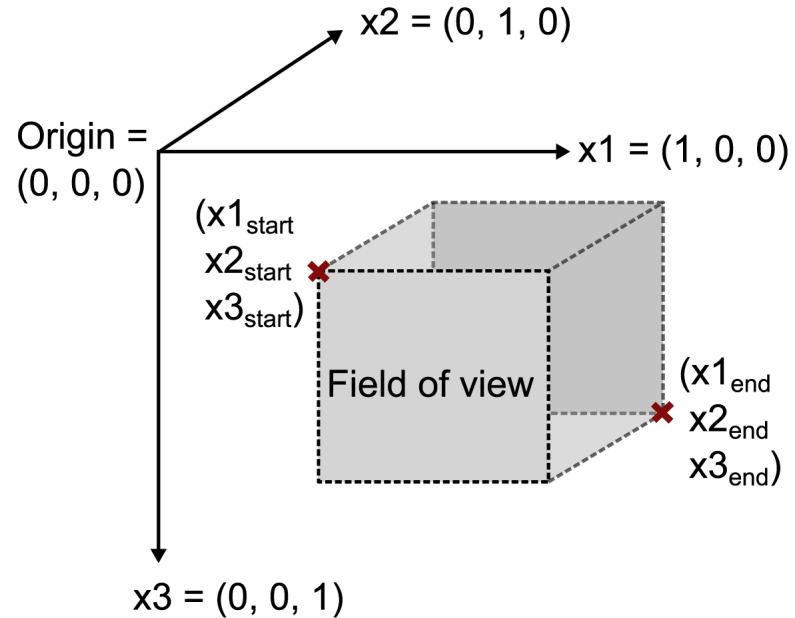
Visualisation of the coordinate system definition used for the standard device definitions.

To represent the spatial configuration of the device, a coordinate system is required. Here, the underlying coordinate system is assumed to meet the following conditions: The axes of the Cartesian coordinate systems are x1 = (1, 0, 0), x2 = (0, 1, 0), and x3 = (0, 0, 1) and the spatial location are given in units of metres [m]. Further conditions are not set, as it is sufficient if all axes definitions remain consistent with respect to the referenced *Device Metadata*. The field of view of the imaging device is given in the same coordinate system with six coordinate points ([x1${}_{\text{start}}$, x1${}_{\text{end}}$, x2${}_{\text{start}}$, x2${}_{\text{end}}$, x3${}_{\text{start}}$, x3${}_{\text{end}}$]) and can be used to normalise the detector and illuminator positions. Thereby, the origin of the imaging system can be defined differently for different types of devices (cf. Fig. 2).

For ease of use, we suggest using the following convention for devices that collect time series data for reconstruction into 2D images:

1. The x1 axis should be defined as the horizontal (lateral) axis of the imaging plane.
2. The x2 axis should be defined as the vertical (axial) axis of the imaging plane.
3. The x3 axis should be defined as the normal (elevation) to the imaging plane.

## 5 PACFISH: An open-source API for data access and conversion

**Fig. 3:**
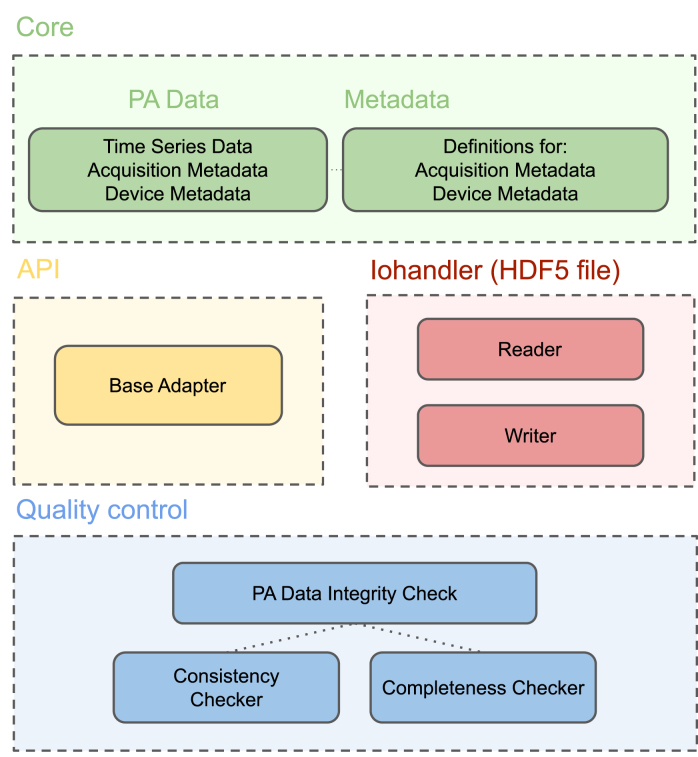
Overview of the software components of PACFISH for Python-based access to the IPASC data format. Different colours represent different modules that encapsulate separate responsibilities of the API: The *api* module is displayed in yellow, the *core* module in green, the *qualitycontrol* module in blue and the *iohandler* in red.

To facilitate the use of the IPASC data format, a prototype Python-based software tool (PACFISH = Photoacoustic Converter for Information Sharing) was implemented (Fig. 3). PACFISH serves three purposes: (1) it helps vendors to integrate the IPASC data format export into their standard software; (2) it assists scientists to read and write data in the consensus HDF5 format; and (3) it helps the PAI community to create custom adapters that convert proprietary file formats into the consensus HDF5 format. PACFISH is available open-source on GitHub^7^ under the commercially-friendly BSD-3-Clause licence^8^ and contributions to the continued development of PACFISH are welcomed.

PACFISH is divided into the API, core, quality control, and iohandler modules. The API package (**pacfish.api** yellow module) can be used to facilitate the integration of adapters for conversion from arbitrary file formats into the IPASC data format. To create a conversion adapter, a Python representation of (1) the binary data, (2) the acquisition metadata dictionary, and (3) the device metadata dictionary needs to be implemented. The adapter must provide at least the *minimal metadata* as defined in the *Metadata Attributes* section. An option to add additional custom metadata items is also provided.

The core classes (**pacfish.core** green module) represent the metadata and data structure in Python. Each metadatum is described with specific device tags defining the name, data type, necessity and SI unit (if applicable), and setting a value constraint. Basic metadata constraints have been implemented to avoid accidental typos within the values field (e.g. only positive numbers larger than zero are applicable for acquisition wavelengths). If the value is not within the constraints a *TypeError* is raised. Metadatum-specific functions enable easy addition of the values for the specific metadata field.

**Fig. 4:**
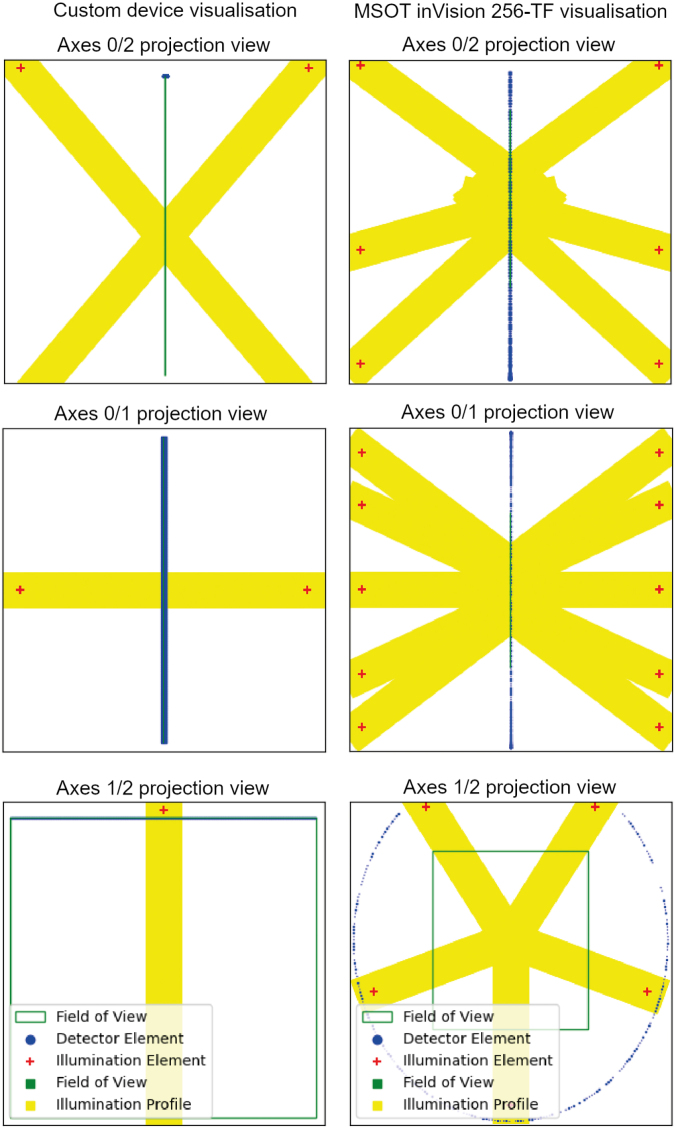
Visualisation of example photoacoustic devices generated from the IPASC metadata. The upper image shows a linear transducer with two illumination slits at either side as described in [19]. The lower image is a representation of the MSOT inVision 256-TF (iThera Medical GmbH, Munich, Germany).

The quality control functionalities (**pacfish.qualitycontrol** blue module) ensure the correctness of the conversion into the IPASC format: a *completeness checker* tests that all metadata are being called and a *consistency checker* ensures that all metadata are within their constraints. An automatically-generated output report gives a human-readable summary of the quality control checks and ensures that the likelihood of conversion mistakes are minimised. To assess the *Device Metadata*, the detector and illuminator positions can be represented in a 3D coordinate system as visual control (Fig. 4).

Finally, the I/O functionality (**pacfish.iohandler** red module) enables reading and writing of IPASC-formatted data files. Code listing 1 shows how to load and access the standardised data and metadata using PACFISH.

**API Usage Examples**

In the following, we provide some example code stubs that can be used to understand the handling of PACFISH, using the version number available at the time of the publication date of this paper. For up-to-date examples, please visit the PACFISH GitHub page.^9^

**1. Using the API to work with data in the IPASC format:**

This listing includes Python code showcasing how to use PACFISH to read data in the IPASC format, to unpack metadata relevant for image reconstruction, and to write data to the hard drive.

**Figure:**
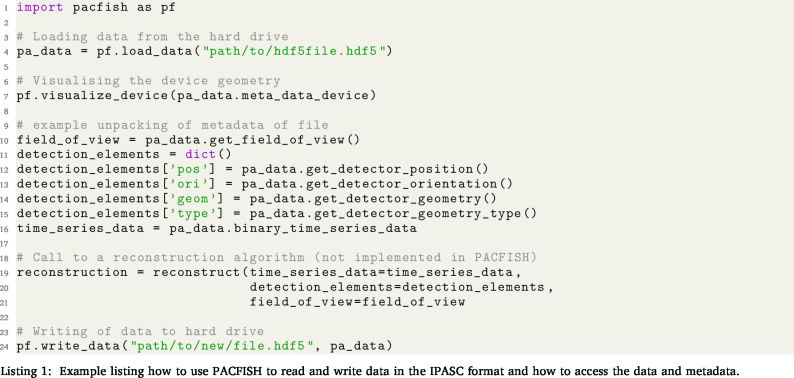

**2. Using the API to write a custom device-specific adapter:**

**Figure:**
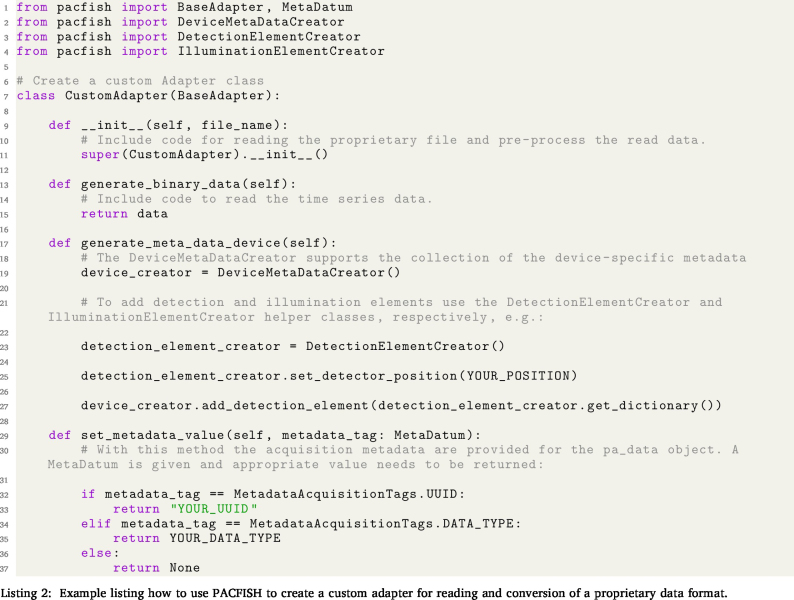

**3. Using the custom adapter to access/convert photoacoustic data:**

**Figure:**
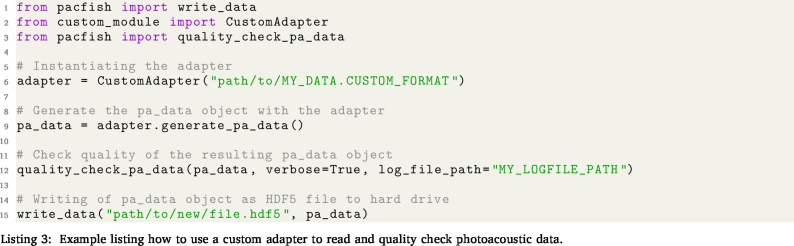

**Fig. 5:**
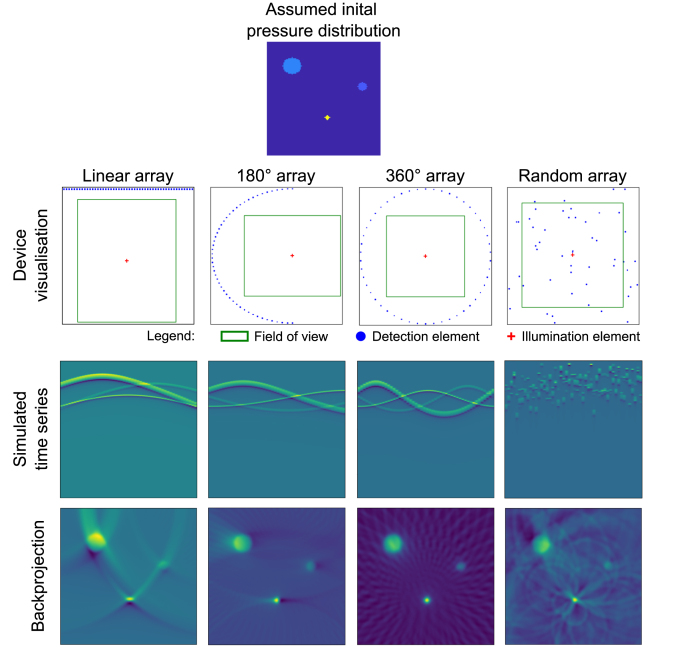
Representative reconstructions of simulated data in the IPASC data format using different detector geometries. The simulated assumed initial pressure distribution is visualised at the top. Device visualisation, simulated time series, and reconstructed data using back projection are visualised underneath in the top, middle and bottom row, respectively. Four different detection arrays have been used: Linear array (first column), semi-circular (180°) array (second column), full circular (360°) array (third column), and a random array (fourth column) .

Sample data in the IPASC format can be found on Zenodo,^10^ and application examples can be accessed on Github.^11^ Fig. 5 shows the Zenodo sample data, displaying the device visualisation, simulated time series data, and reconstruction result of a conventional back projection algorithm [20] for four different sample data sets with different detection geometries.

## 6 Discussion

We have presented the IPASC data format for PAI data storage. The IPASC format is designed to store raw time series data with associated metadata as HDF5 files. To facilitate the use of the IPASC data format, a Python-based software tool (PACFISH = Photoacoustic Converter for Information Sharing) has been created and tested by members of the DAM working group; it is provided to the PAI community to use and further develop.

PAI systems can have vastly different illumination and detection geometries, including linear array, cylindrical, and hemispherical designs. For any hardware configuration, the scanning protocol can also vary (full-scan vs. composite-scan). While the IPASC format is designed to accommodate all needs, limitations remain.

First, the IPASC data format does not yet support the inclusion of data from bimodal systems, such as combined photoacoustic and ultrasound systems. Future implementations should account for these hybrid system types, as they become increasingly common (cf. e.g. [21], [22]), especially in clinical settings.

There are also limitations associated with the use of the underlying data format HDF5, such as the risk of corruption or potential performance issues when dealing with large datasets [23]. Whilst these drawbacks are currently outweighed by the advantages of HDF5 – such as its flexibility, organisation, open access, and its capacity to store large annotated datasets within a single file – the limitations should be kept in mind to ensure safe use of the format.

The IPASC data format is designed to accommodate raw time series measurements with all relevant metadata. Storing raw data is preferable to storing reconstructed images, as reconstruction can lead to image artefacts and loss of information. For reconstructed images, compatibility with the DICOM format is desirable, as it is the most widely used format in management of medical image data. The IPASC industry board, which includes vendors from commercial PAI systems, is currently working towards integrating photoacoustic image data into DICOM. As the DICOM tags will be based on the metadata naming convention presented here, IPASC hopes to enable appropriate mapping between the two formats and to ensure their compatibility.

The PACFISH Python tool is still under development and is currently being tested by members of the photoacoustic community and by vendors to assess its applicability in an industrial setting. Adapters to other programming languages, such as MATLAB, could be added to increase the accessibility of the PACFISH within the user community. Moreover, the direct integration of PACFISH into software tools relevant for numerical forward modelling, such as k-Wave [24] or Monte Carlo eXtreme (MCX) [25] would be beneficial.

Our focus in the near future will be to widen the adaptation of the IPASC format for both the vendor and research communities. Besides implementing additional adapters to proprietary formats, programming languages and software tools, we would like to make established image reconstruction algorithms compatible with the IPASC format to streamline post-processing pipelines using the format. A further aim is to create an open-access database that includes data held in the IPASC format, to facilitate reproducibility studies and structured evaluation and benchmarking of reconstruction algorithms. By introducing the IPASC format, we hope to facilitate technological advancement, foster community collaboration, and ultimately accelerate clinical translation and adoption of this modality.

This article serves only as an introduction to the IPASC data format. The *Agreed Proposal* consensus document [12] with a complete description of the IPASC data format can be found on the IPASC website: https://www.ipasc.science/, which also contains further resources and guidance on how to provide feedback on the documents.

## CRediT authorship contribution statement

**Janek Gröhl:** Writing – original draft, Software. **Lina Hacker:** Writing – original draft, Software. **Ben T. Cox:** Writing – review & editing, Supervision. **Kris K. Dreher:** Writing – review & editing, Software. **Stefan Morscher:** Writing – review & editing, Supervision. **Avotra Rakotondrainibe:** Writing – review & editing, Software. **François Varray:** Writing – review & editing, Software. **Lawrence C.M. Yip:** Writing – review & editing, Software. **William C. Vogt:** Writing – review & editing, Supervision. **Sarah E. Bohndiek:** Supervision.

## Declaration of Competing Interest

One or more of the authors of this paper have disclosed potential or pertinent conflicts of interest, which may include receipt of payment, either direct or indirect, institutional support, or association with an entity in the biomedical field which may be perceived to have potential conflict of interest with this work. For full disclosure statements refer to https://doi.org/10.1016/j.pacs.2022.100339. Sarah Bohndiek reports a relationship with EPFL Center for Biomedical Imaging that includes: speaking and lecture fees. Sarah Bohndiek reports a relationship with PreXion Inc that includes: funding grants. Sarah Bohndiek reports a relationship with iThera Medical GmbH that includes: non-financial support. Avotra Rakotondrainibe reports a relationship with iThera Medical GmbH that includes: employment. Stefan Morscher reports a relationship with iThera Medical GmbH that includes: employment.

## Footnotes

4 MATLAB. The MathWorks Inc., Natick, Massachusetts, 2020.

5 http://h5cpp.org/ (accessed 03/03/2022).

6 https://portal.hdfgroup.org/display/HDF5/HDF5+Java+Documentation (accessed 03/03/2022).

7 https://github.com/IPASC/PACFISH (accessed 03/03/22).

8 https://opensource.org/licenses/BSD-3-Clause (accessed 03/03/22).

9 https://github.com/IPASC/PACFISH (accessed 03/03/22).

10 https://zenodo.org/record/5938838 (accessed 03/03/22).

11 https://github.com/IPASC/PACFISH/tree/main/examples (accessed 03/03/22).
